# Identifying Cattle Breed-Specific Partner Choice of Transcription Factors during the African Trypanosomiasis Disease Progression Using Bioinformatics Analysis

**DOI:** 10.3390/vaccines8020246

**Published:** 2020-05-23

**Authors:** Abirami Rajavel, Felix Heinrich, Armin Otto Schmitt, Mehmet Gültas

**Affiliations:** 1 Breeding Informatics Group, Department of Animal Sciences, Georg-August University, Margarethe von Wrangell-Weg 7, 37075 Göttingen, Germany; abirami.rajavel@uni-goettingen.de (A.R.); felix.heinrich@uni-goettingen.de (F.H.); armin.schmitt@uni-goettingen.de (A.O.S.); 2 Center for Integrated Breeding Research (CiBreed), Albrecht-Thaer-Weg 3, Georg-August University, 37075 Göttingen, Germany

**Keywords:** Animal African Trypanosomiasis, Boran, N’Dama, trypanotolerance, susceptibility, PC-TraFF, transcription factor

## Abstract

African Animal Trypanosomiasis (AAT) is a disease caused by pathogenic trypanosomes which affects millions of livestock every year causing huge economic losses in agricultural production especially in sub-Saharan Africa. The disease is spread by the tsetse fly which carries the parasite in its saliva. During the disease progression, the cattle are prominently subjected to anaemia, weight loss, intermittent fever, chills, neuronal degeneration, congestive heart failure, and finally death. According to their different genetic programs governing the level of tolerance to AAT, cattle breeds are classified as either resistant or susceptible. In this study, we focus on the cattle breeds N’Dama and Boran which are known to be resistant and susceptible to trypanosomiasis, respectively. Despite the rich literature on both breeds, the gene regulatory mechanisms of the underlying biological processes for their resistance and susceptibility have not been extensively studied. To address the limited knowledge about the tissue-specific transcription factor (TF) cooperations associated with trypanosomiasis, we investigated gene expression data from these cattle breeds computationally. Consequently, we identified significant cooperative TF pairs (especially DBP−PPARA and DBP−THAP1 in N’Dama and DBP−PAX8 in Boran liver tissue) which could help understand the underlying AAT tolerance/susceptibility mechanism in both cattle breeds.

## 1. Introduction

Climate change is likely to increase the risk of several vector-borne diseases including human and animal Trypanosomiasis [1]. African Animal Trypanosomiasis (AAT), also known as nagana disease, is a chronic parasitic infection which affects livestock in large numbers, prevalently found in the cattle of sub-Saharan Africa [2,3]. Trypanosomes are unicellular protozoans, which are transmitted through the saliva of the vector tsetse fly. They survive in the bloodstream of the animal by escaping and manipulating the host’s immune response, thereby causing serious health problems in cattle [4]. Due to this debilitating infection, the animal becomes diseased which results in lower economic productivity such as reduced meat and milk production and reduced draught power for agricultural production, thus imposing huge financial losses to farmers in sub-Saharan Africa [5]. Especially the main causative species *Trypanosoma congolense* and *Trypanosoma vivax* severely impair the health of the cattle population [6,7].

The animals display numerous clinical signs in the early and later stages of the disease with anaemia being the most prominent pathological feature of AAT [8]. Other clinical signs include fever, intermittent chills, weight loss, lethargy, emaciation, neurological disorders, infertility, abortion, difficulty in breathing, loss of appetite, and congestive heart failure leading to death if left untreated [2,3,5,9].

Some cattle breeds are capable of resisting the disease [after trypanosome infection] despite the parasite’s infection in the body. Those cattle breeds are known to be resistant to trypanosomiasis and this trait is termed as trypanotolerance. Trypanotolerance is a distinctive trait of few West African taurine breeds, which has been gained through natural selection by continuous interaction of host and parasite [10,11]. One such trypanotolerant *Bos taurus* cattle breed is N’Dama [12]. Even though N’Dama cattle are trypanotolerant, they are not particularly advantageous for agricultural purposes because of their low productivity and their small size. Whereas the other cattle breed Boran is beneficial for their productivity, heat tolerance and performance, it is highly susceptible to trypanosomiasis [13]. Therefore, understanding the gene regulatory mechanisms underlying the biological processes for their susceptibility/resistance is useful for the selective breeding of this trait.

Trypanosomes are able to escape the host’s natural and adaptive immunity due to their antigenic variation [14,15,16,17]. This speculative adaptation of the parasite is of great interest in recent times because of the existing and increasing risk of drug resistance to the currently available trypanocides [18,19]. Henceforth, there is an urgent need for the understanding of the molecular mechanism underlying this infectious tropical disease.

For this purpose, Noyes et al. [20] performed gene expression analysis of a transcription profiling time series microarray dataset for liver, spleen and lymph node tissues in the cattle breeds Boran and N’Dama. These tissues are vital lymphoid organs which mount host immune responses to pathogenic invasion by generating high numbers of macrophages involved in phagocytosis and further production of pro-inflammatory cytokines [21,22,23]. By analysing this dataset, Noyes et al. identified the differentially expressed genes (DEGs) based on which the candidate genes within previously reported QTLs in the regulation of immune responses were obtained. However, according to its clinical signs, AAT could be considered as a multistage progression process. While mainly focusing on the DEGs, Noyes et al. studied the differences in expression levels at certain timepoints, but their analysis strategy could not capture the importance of the genes with regular monotonic expression patterns as the disease advances. Recently, Wang et al. [24] and Suyan Tian [25] have pointed out that the genes with monotonic expression patterns are quintessential for gaining complete insight into the multistage progression of the disease.

Despite the rich literature on trypanotolerance and molecular studies addressing the identification of candidate markers in AAT, its underlying molecular mechanism in cattle has not been well studied. As of now, there has been no study performed in AAT especially in cattle, with the aspect of examining the role of the regulatory elements involved in gene regulation, for example, TFs and their cooperations. Today it is well known that TFs and their complex interplay have critical roles in the progression of disease [26,27]. In order to address the importance of cooperative TFs in the AAT disease, we analysed in this study the dataset published by Noyes et al. [20]. Unlike this study, we focus on the identification as well as the analysis of monotonically expressed genes (MEGs) to completely capture the multistage progression process of the AAT disease. Further, we applied our previously published computational PC-TraFF approach [26] to the promoter regions of the MEGs in order to identify specific cooperative TFs in different tissues of Boran and N’Dama cattle breeds. Our results suggest that the preferential partner choice of TFs could be related to the gene regulatory mechanisms determining the level of AAT-tolerance of the cattle breeds. Particularly with regard to AAT-disease, the partner choice of the transcription factor *DBP* is likely to orchestrate the genetic programs governing the molecular mechanism of the level of trypanotolerance of both cattle breeds. Especially, focusing on DBP’s function in association with the circadian rhythm, we attempted to highlight the significant role of the circadian transcriptional program in regulation of immune responses to the pathogen infection at the tissue level (see the review [28] for details regarding the circadian regulation of immunity).

### 1.1. Conserved Functions of Transcription Factors across Mammals

Transcription factors (TFs) are proteins which bind to short DNA sequences known as Transcription Factor Binding Sites (TFBSs), involved in regulating the transcription of genes [26,29,30]. These two interacting regulatory elements (TFs and TFBSs) are two of the significant functional elements which are involved in the regulation of various cellular processes [29,30]. According to the widely accepted phylogenetics footprinting approach, the basic assumption is that the functional elements are likely to be more conserved than the non-functional elements in response to selective pressure [31,32]. Therefore, the functions of transcription factors and the binding sites are expected to be well conserved across multi-species, particularly across evolutionarily closely related mammalian species. Several studies also confirm the evolutionary conservation of binding specificities of TFs in a wide range of species [33,34,35,36]. In our study, the predictions are performed for the tissues of two different cattle breeds. Regarding the conserved functions of TFs across mammals, we interpreted the results for cattle breeds based on experimental studies which have been designed and performed for other mammalian species (such as model organisms including human and mouse).

### 1.2. Transcription Factors, Potential Targets for Vaccine Development

Transcription factors play an important role in mounting immune responses especially during pathogenic invasion [37,38]. Both innate and adaptive immunity of the immune system are controlled at the transcriptional level, which thereby provides valuable drug targets for regulating the gene expression of several immune cells [39]. Almost 10% of anti-cancer drugs approved by Food and Drug Administration (FDA) targets the transcription process [40]. Recently, transcription therapy has been proposed as a state-of-the-art approach targeting transcription factors for therapeutic interventions [41]. Therefore, transcription factors, being converging point for many signalling pathways, can serve as promising drug candidates for vaccine development [42]. In our study, we focused on transcription factors and their complex interplay which orchestrate the genetic programs underlying the level of trypanotolerance of both cattle breeds. Our findings could provide novel drug targets for the development of effective vaccine-mediated control of the AAT disease.

## 2. Materials and Methods

**Figure 1:**
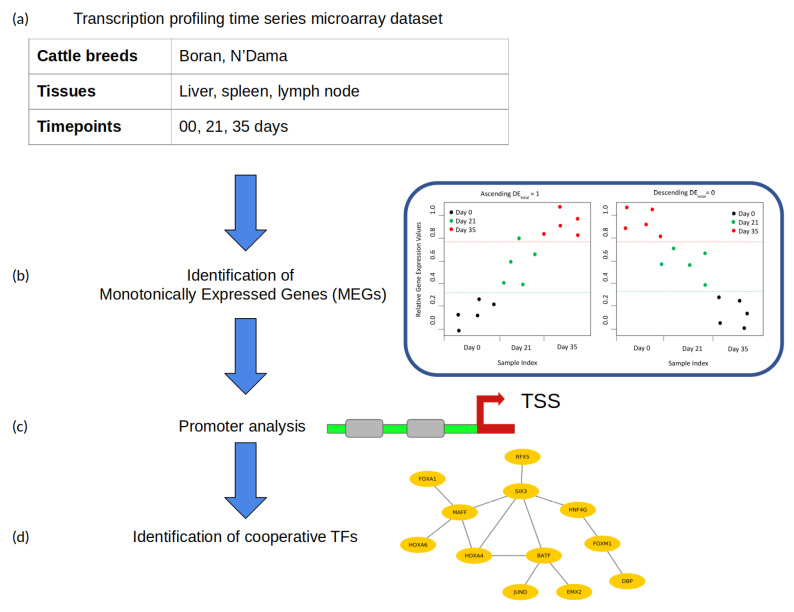
Flowchart of analysis. (**a**) Processing of the microarray dataset; (**b**) identification of Monotonically Expressed Genes using the MFSelector approach; (**c**) promoter analysis (TSS: transcription start site); (**d**) identification of cooperative TFs using PC-TraFF approach.

In this section we describe the microarray gene expression dataset that we analyzed and the methods applied in this study. Our analysis follows the structure as represented in Figure 1.

### 2.1. Microarray Dataset

A microarray experiment was undertaken by Noyes et al. (http://www.ebi.ac.uk/arrayexpress/, accession no. E-MEXP-1778) [20] to survey the genome of two cattle breeds for differentially expressed genes and their related pathways in different tissues. The dataset contains the gene expression obtained from a trypanosomiasis-susceptible (Boran) breed and a trypanosomiasis-resistant (N’Dama) breed at different time points (days: 0, 12, 15, 18, 21, 26, 29, 32 and 35) for three tissues, namely liver, spleen and lymph nodes. In their study, Noyes et al. [20] included 25 trypanosomiasis-free (healthy) animals of each breed which were chosen from herds at a tsetse fly free- and trypanosomiais-free zone of the ILRI Kapiti Plains ranch. Furthermore, before being transferred to the ILRI research facility at Kabete, they were tested for tick-borne parasites and confirmed negative. Afterwards, all animals were infected with *T. congolense*, which is one of the infectious species causing trypanosomiasis. N’Dama was selected for its trypanotolerance and Boran for its trypanosusceptibility. At different time points after infection, the tissues were collected from the animals. For control experiments, the tissue collection was undertaken similarly for the two breeds before infection and noted as day 0. Tissues from liver, spleen and lymph nodes were harvested on days 0, 21 and 35 after infection. Furthermore, liver tissues at additional time points on days: 12, 15, 18, 21, 26, 29 and 32, were collected by biopsy. RNA from each tissue was extracted and hybridised on arrays. Consequently, the dataset altogether consisted of 160 samples from three cattle tissues, containing expression values for 13,934 cattle genes. The expression strength is given in transcript per million (TPM) values.

### 2.2. Identification of Monotonically Expressed Genes

We applied the monotonic feature selector (MFSelector) approach [24] in order to identify the genes with a strong monotonic pattern (either in ascending or descending order) in their expression profiles over time points during disease progression.

The MFSelector approach requires gene expression datasets measured over several time points as input and assesses the confidence of the monotonicity of each gene by calculating its total discriminating error (DE${}_{\mathrm{total}}$).

Let $g_{ijs}$ be an expression value of gene $g_{i}$ measured at time point j=1,⋯,T from sample s=1,⋯,S. The discriminating error (DE) of $g_{i}$ is calculated by comparing its expression values $g_{ijs}$ observed for all samples *S* at time point *j* against the remaining time points as:

(1) $$\begin{array}{c} \mathrm{DE}\left(g_{ijs}\right)=\left\{\begin{array}{cc} 1 & \;\mathrm{if}\;\;\;\;g_{ijs}>\tau_{j}\\ 0 & \;\mathrm{otherwise}\; \end{array}\right. \end{array}$$

In Equation (1), $\tau_{j}$ refers to the threshold for discriminating line which distinguishes in ascending order the $g_{ijs}$ values of all samples at time point *j* from other time points and simultaneously ensures the minimum total discriminating error for *j*. Consequently, the DE${}_{\mathrm{total}}$ score for $g_{i}$ is calculated as:

(2) $$\begin{array}{cc} {\mathrm{DE}}_{\mathrm{total}}\left(g_{i}\right)= & \sum_{j=1}^{T}\sum_{s=1}^{S}\mathrm{DE}\left(g_{ijs}\right) \end{array}$$

Similar as in Equation (1), the discriminating error of genes is calculated to assess their monotonicity in descending order as:

(3) $$\begin{array}{c} \mathrm{DE}\left(g_{ijs}\right)=\left\{\begin{array}{cc} 1 & \;\mathrm{if}\;\;\;\;g_{ijs}<\eta_{j}\\ 0 & \;\mathrm{otherwise}\;, \end{array}\right. \end{array}$$

where $\eta_{j}$ is the threshold for the discriminating line at *j* and lastly the DE${}_{\mathrm{total}}$ score is calculated again using Equation (2).

In the next step, MFSelector performs a permutation test for the assessment of the statistical significance of the ${\mathrm{DE}}_{\mathrm{total}}$ scores by calculating their unadjusted *p*-values and *q*-values which were adjusted *p*-values for multiple testing. Finally, based on the Sample Variance for Discriminating Error (*svde*), the level of confidence of a monotonic gene is determined. A small *svde* value indicates clear monotonicity of the corresponding gene.

For the application of the MFSelector package, we have to define parameters such as *permut*, *svdetimes* and *svdenoise* in order to set the level of stringency for the monotonicity. *permut* controls the statistical significance of the ${\mathrm{DE}}_{\mathrm{total}}$ index, *svdetimes* represents the number of times the SVDE procedure along with the random noise will be repeated and *svdenoise* indicates the strength of the noise in the experiment. The greater the values of the aforementioned parameters, the higher the stringency of the selection. For our analysis, to obtain the MEGs which are expressed with strong monotonic pattern, we applied the MFSelector approach by setting its parameters: *permut* 100, *svdetimes* 100 and *svdenoise* 0.1.

### 2.3. Identification of Transcription Factor Cooperation

In order to identify cooperative transcription factors, we applied our previously developed PC-TraFF method [26] which is a well established information theory based approach. The PC-TraFF algorithm uses the concept of pointwise mutual information (PMI) for the identification of TF cooperation by mainly considering the co-occurrence of their transcription factor binding sites (TFBSs) in the promoters of genes [26].

As input parameters the algorithm needs a set of regulatory sequences, a library of position weight matrices (PWMs) and pre-defined TFBS distance constraints.

- **Promoter sequences:** The promoter sequence (covering the −500 to 100 bp regions relative to a transcription start site) of each significant monotonically expressed gene (MEG) is extracted from the UCSC genome browser [43].
- **Creation of the PWM library and TFBS detection:** For the detection of TFBSs in the promoters of MEGs, we obtained PWMs from the TRANSFAC database (release 2018.1) [44].
  Until now, based on the functional analysis and comprehensive performance evaluation strategies, different studies have shown that the computational TFBS detection methods using PWMs are well established and highly applied. However, their prediction performance is prone to high rates of false positive predictions [30,45,46]. In order to eliminate the false predictions to some extent in our analysis, we manually created a specific PWM library following our previous study [47]. For this purpose, we first obtained all available cattle TFs from AnimalTFDB 2.0 [48] and identified the expression values (TPM values) of their corresponding TF genes in the gene expression dataset, under study. Second, the TFs were excluded from further analysis if the TPM values of their TF genes were zero. After that, the corresponding PWMs of the remaining TFs were obtained from the TRANSFAC database [44]. Finally, based on the Pearson correlation between these PWMs, we applied hierarchical clustering and used only the PWMs with the highest information content from each cluster as representative to create our final non-redundant vertebrate PWM library (see Supplementary Table S1).
  In addition, we applied the Match${}^{\mathrm{TM}}$ program [49] using these PWMs and their TRANSFAC specific profile parameter *minSum* to minimize the sum of false positive and false negative rate for the detection of putative TFBSs in promoter sequences.
- **Pre-defined distances:** For the identification of cooperative TFs based on the co-occurrence of their TFBSs, the PC-TraFF algorithm requires pre-defined minimum and maximum distance thresholds. In this study, the recommended distance values of ≥5 and ≤20 were used for the minimum and maximum distance, respectively.

The PC-TraFF algorithm provides a PMI$\left(T_{a};T_{b}\right)$-score for each cooperative TF-pair ($T_{a}$ and $T_{b}$), which is transformed in the next step into the *z-score*. A cooperation between any $T_{a}$ and $T_{b}$ is considered to be statistically significant if they have a *z-score*≥3.

## 3. Results and Discussion

### 3.1. Data Processing

We analysed a time series dataset which consisted of gene expression values for three tissues (liver, spleen and lymph node) from two cattle breeds, trypanosusceptible and trypanotolerant, during the disease progression after *T. congolense* infection. Although the dataset for liver tissue consisted of gene expression values from several time points (days 0, 12, 15, 18, 21, 26, 29, 32 and 35), we considered for this tissue the data only for 3 time points (days: 0, 21 and 35) similar to spleen and lymph node tissues, to ensure the purpose of maintaining uniformity throughout the analyses.

### 3.2. Identification of MEGs

To begin with the analysis, we organized the dataset for each breed separately and arranged the dataset unique for the tissue type of each breed in ascending order of time points. Afterwards, we applied the MFSelector package to the gene sets of each tissue, using its parameters as mentioned in the Materials and Methods section. Subsequently, we obtained for each tissue in both cattle breeds two lists of MEGs in ascending and descending order based on their monotonicity of expression during the disease progression. Finally, we defined for our further analysis a gene to be a statistically significant MEG if its corresponding *q*-value ≤ 0.05 and its *svde* value ≤ 1. The numbers of statistically significant MEGs are given in Table 1 and all lists of MEGs are provided in Supplementary Table S2.

**Figure 2:**
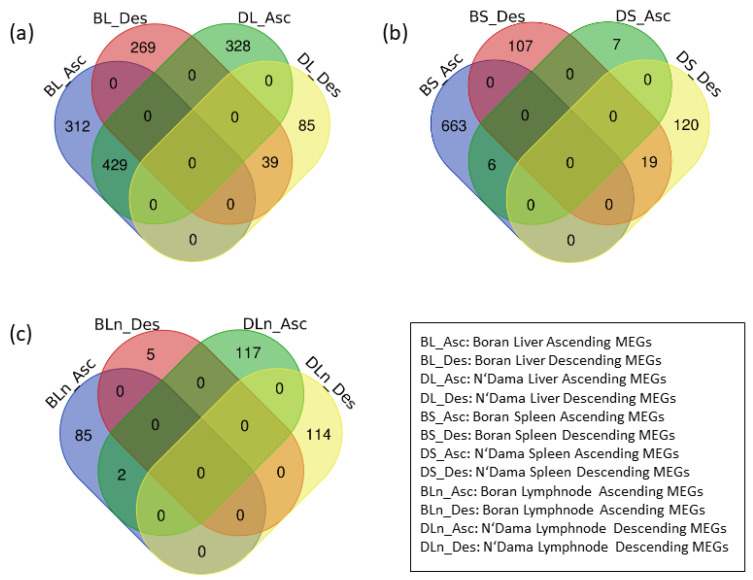
Venn-diagram of the MEGs in the ascending and descending orders of (**a**) liver-, (**b**) spleen-

The analysis of significant ascending and descending MEGs for spleen and lymph node tissues revealed that the vast majority of them are unique in both breeds and only a small minority of these MEGs is common to both breeds. On the other hand, there are 429 overlapping genes in the significant ascending MEGs and 39 overlapping genes in the descending MEGs in the liver tissue of Boran and N’Dama, respectively (see Figure 2). In our further analysis, we omitted the MEGs which are found to be significant and overlapping for a certain tissue in both breeds.

### 3.3. Identification of Cooperative TFs

Applying the PC-TraFF approach [26] to the promoters of MEGs for each tissue in both cattle breeds individually, we have identified significant cooperative TF pairs for liver, spleen and lymph node tissues which are listed in Table 2. The TFs *E2F1*, *PPARA*, *THAP1*, *HAND1E47* and *TFAP2A* are frequently observed in all three tissues of both cattle breeds. The factor *E2F1* belongs to the E2F family of transcription factors [50] and is involved in promoting the process of adipogenesis and in regulating lipolysis [51,52]. Denechaud et al. pointed out the association of *E2F1* with several processes in pancreas, liver, heart, muscle, and adipocytes including lipogenesis, cholesterol transport, bile acid synthesis, glucose oxidation, and oxidative metabolism [52]. Furthermore, it was also reported that *E2F1* mRNA levels in the adipose tissue correlated with circulating free fatty acid levels in obese human subjects [52,53]. Interestingly, E2F has been reported as one of the promising candidates in the circadian transcriptional regulators [54].

Kersten et al. explained in their review [55] *PPARA* is activated by ligands and is abundantly found in liver. In mouse, it is reported as the master regulator of lipid metabolism in liver during fasting. In human liver, it is reported that *PPARA* induces several genes involved in numerous metabolic pathways including bile acid synthesis, lipoprotein metabolism, synthesis and breakdown of triglycerides and lipid droplets. Moreover, they play suppressive roles in inflammation and acute phase response.

The factor THAP domain containing apoptosis associated protein 1 abbreviated to *THAP1* belongs to the THAP protein family [50,56,57]. They are found in the interaction and co-localization with the promyelocytic leukemia nuclear bodies [56]. Another important factor is *TFAP2A* which is a member of the *TFAP2* (AP-2) family of basic helix-span-helix transcription factors. A crucial role of these transcription factors has been shown in [58] as the master regulator of lipid droplet biogenesis, in which lipid droplets are known for various other functions including inflammatory responses, host-pathogen interaction, and other metabolic processes [58,59,60]. Importantly, it was found to be significantly over-represented in the promoter regions of clock controlled genes [54].

On the other hand, the factor *HAND1E47* from the basic helix-loop-helix (bHLH) transcription factor family is reported in the development of heart [61,62,63,64] and to be associated with cardiac defects [65]. Its association with the host pathogen interplay is currently biologically unconfirmed.

**Figure 3:**
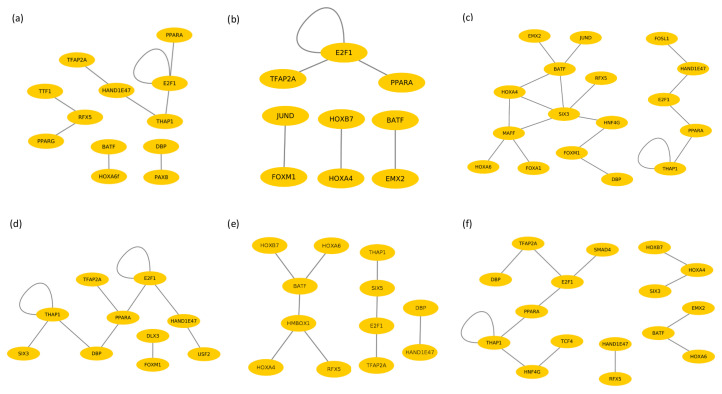
Cooperation networks for the TF pairs of (**a**) liver tissue of Boran, (**b**) spleen tissue of Boran, (**c**) lymph node tissue of Boran, (**d**) liver tissue of N’Dama, (**e**) spleen tissue of N’Dama and (**f**) lymph node tissue of N’Dama.

In order to gain a better understanding of the underlying molecular mechanism of AAT in different tissues and compare the results of both breeds, we created cooperation networks for each tissue based on its specific TF pairs as suggested in our previous studies [26,47,66,67]. The nodes represent the TFs and the edges represent their co-operation in these networks which are presented in Figure 3. The cooperation networks of liver-, spleen- and lymph node tissues consist of 9, 6 and 18 cooperative TF pairs in Boran and 10, 9 and 13 pairs in N’Dama, respectively.

### 3.4. Cooperative TFs in Liver Tissue

The analysis of the cooperation networks for liver tissue (Figure 3a,d) reveals that, although several (single) TFs are overlapping in both networks, they change their partners in both breeds. Among others, cooperation of the factor *DBP* in these two cattle breeds highlights the difference in its remarkable preferential partner choice, namely *DBP–PAX8* in Boran, whereas *DBP–THAP1* and *DBP–PPARA* in N’Dama.

The albumin Site D-binding Protein (*DBP*) is regarded as a clock target gene which regulates primarily the sleep-wake cycle in mammalian species [54,68,69,70,71]. Further, *DBP* is associated with the circadian rhythm [68], which is normally disrupted in humans during the disease progression [72]. In Human African Trypanosomiasis (HAT), an improper disorganised sleeping pattern is common wherein the infected persons sleep more during day time and stay awake during night time [72]. The relationship between clock genes and the circadian rhythm are well established in several studies [73,74,75]. Further, *DBP* is a liver specific transcriptional activator, expressed in a circadian manner [76]. Circadian rhythm in peripheral tissues like liver is crucial for the normal hepatic metabolism [77,78], especially of lipids [79]. Several binding sites for *DBP* are reported in the promoter regions of the gene *CYP7* which is involved in the rate limiting step of the pathway converting cholesterol to bile acids in mammalian species [80].

In the liver tissue of the cattle breed Boran, *DBP* forms dimers with the factor *PAX8* which is one of the thyroid-specific transcription factors essential for the development of the thyroid gland [81,82]. Mutations in *PAX8* have been reported to cause hypothyroidism [83]. *PAX8* is a highly sensitive marker for thyroid and renal tumors [84]. The levels of the Thyroid Stimulating Hormone (TSH) are influenced by the circadian rhythm [85]. In relation to AAT, one of the endocrine organs affected by trypanosome infection is thyroid. Interestingly, decrease in the levels of T4 has been observed in goats after *T. congolense* infection, consequently indicating the impairment of the thyroid function [86]. Studies on Boran cattle infected with *Trypanosoma congolense* have confirmed the impairment of the pituitary gland [87].

Using neonatal *Pax8* −/− mice, about a 10-fold increase of accumulation of hepatic triglycerides has been observed [88]. After the administration of thyroid hormone (TH), hepatic triglycerides were mobilised and processed [88]. Thyroid hormones and the functioning of the liver, particularly in the lipid metabolism, are interconnected with each other. Additionally, it is proven that thyroid hormone regulates a variety of metabolic processes by interacting with several important signalling pathways, thereby influencing energy metabolism and energy homeostasis [89]. They critically regulate the cholesterol metabolism in rat [90]. Specifically in liver, T3 and T4 hormones regulate the lipoprotein metabolism [88,91,92]. These findings support the hypothesis that the cooperation between the TFs *DBP* and *PAX8* could be strongly associated with the circadian rhythm, thyroid hormones and the lipid metabolism of the AAT susceptible breed Boran.

On the other hand, the TF cooperations *DBP–PPARA* and *DBP–THAP1* in the liver tissue of N’Dama might be noticeably changing the host and parasite interaction in a direction that is opposite to that of Boran. The factor *PPARA* from the Peroxisome proliferator-activated receptor family is reported to be an important regulator of lipid metabolism, predominantly expressed in liver. It belongs to the nuclear receptor hormone superfamily which are ligand-induced [93]. PPARs are increasingly studied in inflammation as they are involved directly in the negative regulation of inflammation. Remarkably, *PPARA* plays a significant anti-inflammatory role in the regulation of the immune system [93]. The second partner of *DBP* is the zinc fnger transcription factor *THAP1* [56,94,95]. Mutations in *THAP1* result in neuronal dysfunction leading to dystonia, a brain disorder which is characterized by involuntary muscle contractions and abnormal postures [96].

After *T. congolense* infection, cerebral lesions and enlargement of several organs in particular liver, spleen, lungs, heart, and lymph nodes, are observed during the pathogenesis of the disease [97,98]. This could be an indication that the infected cattle are attempting to remove the parasites from the body via chronic inflammation. Preferential partner choice of the factor *DBP* in the liver of both breeds could play an influential role in their AAT-tolerance mechanisms. In a study performed by Kierstein et al. [99] in mouse models infected with *T. congolense*, *DBP* has been identified as one of the Differentially Expressed Genes (DEGs) between susceptible and tolerant mice [99]. In a similar study, genes related to lipid transport and metabolism are frequently reported during the progressive stages of the disease [99]. Contrarily in N’Dama, the cooperation between *DBP–PPARA* could be leading to the regulation of lipid metabolism and inflammation. The parasitic trypanosomes might have altered the aforementioned regulatory mechanism of the host by changing the TF cooperation, especially that of *DBP*. This might be the hidden link between metabolic and immune system related pathways. In Boran liver, the *DBP-PAX8* cooperation might be favouring the survival of the parasite in manipulating the pathways for lipid metabolism, which are essential for the parasite. On the other hand in N’Dama, strong transcriptional regulation of metabolism and inflammation might be serving as a critical switch in AAT-tolerance. Regarding the function of *DBP* in controlling the circadian rhythm of liver tissue in mammals and its relation to the AAT disease, our findings suggest that the specific partners of *DBP* in both breeds could be associated with different genetic programs governing their susceptibility or tolerance.

Another interesting TF found in the liver tissue of Boran is *RFX5*. The factor *RFX5* belongs to the family of *RFX* (Regulatory Factor X) gene transcription factors [50] and its over-expression has been observed in hepatocellular carcinoma [100]. Previous studies have shown its critical importance in the regulation of the MHC (Major Histocompatibility Complex) class II gene for which *RFX5* activates the expression of those genes essential for the initiation and propagation of the antigen-specific immune T cells [101,102,103]. In this regard, MHC II genes are shown to play an important role in the adaptive immunity [101,102,103]. In the thymus, they are important for the positive and negative selection of T-cells [101,102,103].

In the liver tissue of Boran, the *RFX5* cooperates with the factors *TTF1* and *PPARG* (see Figure 3a). The factor *TTF1* (Thyroid Transcription Factor 1) is a nuclear protein expressed in the thyroid and the pulmonary epithelium [104,105]. It serves as a specific marker for lung and rectal adenocarcinoma [106,107,108]. Together with the factor *PAX8*, the factor *TTF1* is a particularly important player in the organogenesis of the thyroid gland; both are reported in several studies of thyroid carcinomas [109,110,111]. The second cooperation partner of *RFX5* is *PPARG*, which is a nuclear receptor with anti-inflammatory role and it contributes to cardiovascular diseases [112]. Furthermore, it regulates the expression of *CD36* upon induction, which is involved in processes such as angiogenesis and inflammation. *PPARG* acts as modulator in adipogenesis, insulin sensitivity and the whole-body lipid metabolism [113,114,115,116].

On the other hand, the cooperation network of liver of N’Dama contains the transcription factors *USF2* and *FOXM1* which are strongly associated with immune responses (Figure 3d). Upstream Stimulatory Factor (*USF2*) is a member of the basic helix-loop-helix family that has been identified as one of the controllers of insulin synthesis [117]. Further, *USF2* participates in the regulation of important cellular processes like metabolism, embryonic development, brain function, fertility, iron homeostasis and immune responses [118,119]. In a breast cancer study, levels of *USF2* were reported abnormal and were suggested to play a role in cancer progression [120].

The factor *FOXM1* belongs to the Forkhead box (FOX) transcription factor family [50] and is involved in a variety of biological processes including DNA damage response, drug resistance, cell death, and cell proliferation [121]. Furthermore, *FOXM1* is regarded as the master regulator for DNA damage response and genotoxic agent resistance [122,123]. Based on its regulatory role, it is studied as a potential target for prognosis and treatment of cancers [124].

Taken together, the cooperative TF pairs in the liver tissue of both breeds could provide promising information to elucidate their regulatory genetic programs governing their susceptibility and tolerance traits.

### 3.5. Cooperative TFs in Spleen Tissue

Examining the cooperation networks for the spleen tissue of Boran and for N’Dama (Figure 3b,e) illustrates that all single TFs have different partners in the two breeds except for the TF pair *E2F1–TFAP2A*. Interestingly, the factor *DBP* forms only one dimer in the spleen tissue of N’Dama and it is absent in the cooperation network of Boran spleen tissue. The absence of *DBP* might be indirectly a significant implication of the disruption of the circadian rhythm and the related rhythmic processes in the spleen tissue as a result of trypanosome infection in Boran.

In contrast to Boran, the factor *DBP* forms a cooperative pair with the factor *HAND1E47* in the TF network of the spleen tissue of N’Dama. Taking into account the significance of the interaction between *DBP* and *HAND1E47*, the factor *HAND1E47* belongs to the bHLH transcription factor family, which is mainly involved in the cardiogenesis and hematopoiesis processes, as per the studies in Drosophila model [125]. Furthermore, loss-of-function mutation in *HAND1* has been reported in dilated cardiomyopathy, which is the continuous enlargement and loss of contraction of the ventricular chamber in the heart [126]. The *DBP-HAND1E47* cooperation in the spleen tissue seems to have great importance in the AAT disease resistance of N’Dama, because the AAT-infected cattle die in the final stage of the AAT disease, from cardiovascular defects wherein the TF cooperation *DBP-HAND1E47* could play a defensive role in N’Dama.

A closer look at the cooperation networks of the spleen tissue further reveals that there are several homeobox transcription factors in both networks. Particularly, the factors *HOXA4* and *HOXB7* are found in the networks of both breeds, however with different TF partners. The TFs *HOXA*, *HOXB*, and *HMBOX1* are involved in the regulation of differentiation of haematopoietic cells [127,128,129,130] and development of the embryo [131]. *HOX* genes have been shown to be master regulators of haematopoiesis and are related to haematopoietic disorders [132]. Consequently, their TF cooperations might also play important roles during the AAT disease as the cattle suffers from anaemia which is the destruction of blood cells. Moreover, *HOXA4* has been reported in relation to Chronic Myeloid Leukaemia (CML) [133] and increased expression of *HOXA6* in Acute Myeloid Leukaemia [134]. Furthermore, the factor *HMBOX1* in the network of N’Dama, functions as a transcriptional repressor of the cell cytolytic activity of NK cells [135]. The factor *HMBOX1* cooperates with *BATF*, *HOXA4*, and *RFX5* only in the network of N’Dama which could lead to a significant difference in the regulatory events of the spleen tissue between Boran and N’Dama. *HMBOX1* regulates the process of cell cytolysis, which could thereby be controlling the destruction of blood cells and maintaining the normal blood count of blood cells. According to its known molecular functions, the factor *HMBOX1* might have a protective role in the spleen tissue against splenomegaly and anaemia which are prominent features of AAT.

Although the factor *BATF* has been identified as significant in both networks, it switches its partner (see Figure 3b,e). *BATF* belongs to the bZIP family of transcription factors and is predominantly expressed in lymphocytes. Its preferential partner choice could be strongly associated with the production of immune responses since this TF is specialized in controlling the differentiation of Th17 cells [136].

Another interesting factor is *SIX5* which is found only in the network of N’Dama. *SIX5* belongs to the Sine Oculis homeobox homolog family and is mainly involved in the process of differentiation, migration, and organogenesis [137]. In mouse, *SIX5*-deficient animals displayed characteristics of myotonic dystrophy [137,138,139], which is characterized by muscle weakness, cataracts, heart conduction complications and impaired cognitive functions [139]. The absence of *SIX5* in Boran could also lead to the different AAT-disease signs.

Collectively, our findings in the spleen tissue of both breeds suggest that the specific partner choice of TFs could potentially contribute to splenomegaly, anaemia and immune responses in the susceptible breed Boran.

### 3.6. Cooperative TFs in Lymph Node Tissue

Analysis of the cooperation networks of the lymph node tissue (Figure 3c,f) demonstrates that few single TFs are common in the networks of both breeds, however, with different partners. In particular, the transcription factor *DBP* forms different dimers in these cooperation networks. Interestingly, in Boran lymph node *DBP* cooperates with *FOXM1*, which is a crucial mediator of inflammatory responses. Further, knocking out *FOXM1* has resulted in the reduction of inflammatory response in osteoarthritis [140], suggesting that *FOXM1* could play a crucial role in chronic inflammation in Boran during the disease. On the other hand, in N’Dama, the cooperation partner of *DBP* is *TFAP2A* which belongs to the *AP2* transcription factors. Surprisingly similar to TFs found in liver tissue, also the TF *TFAP2A* is strongly related to lipid droplet biogenesis, which plays an important role in host-pathogen interaction [58,141]. According to the regulatory functions of *TFAP2A*, the cooperation of *DBP-TFAP2A* could be remarkably in strong favour of the rhythmic regulation of lipid droplet biogenesis process in the trypanotolerant breed N’Dama.

The cooperation *DBP-FOXM1* in Boran could be in relation with the regulation of inflammation processes. In contrast to Boran, the *DBP-TFAP2A* cooperation might be involved in the regulation of the circadian rhythm in lymph node tissues of N’Dama.

Another transcription factor found in the cooperation network of Boran is *MAFF*, a leucine zipper (bZIP)-type transcription factor that cooperates with the following factors: *FOXA1*, *SIX3*, *HOXA4* and *HOXA6*. Remarkably, SNPs in *MAFF* are experimentally reported to be in association with Chronic Myeloid Leukaemia [142]. The symptoms of Chronic Myeloid Leukaemia closely resemble the condition of AAT-affected cattle which suffer from similar weight loss, lymphadenopathy, splenomegaly, hepatomegaly, and cardiac failure during the disease [3,143].

The factor *SMAD4* which is found in the network of N’Dama, is crucial for the regulation of differentiation of Th17 cells. As previously mentioned, Th17 cells are important in the investigation of inflammatory and autoimmune diseases. Furthermore, mutations on *SMAD4* had resulted in the loss of suppression of Th17 cell differentiation and therefore they also serve as therapeutic target for autoimmune disorders [144]. As shown in the network of Boran, *BATF*, and *JUN* complexes have been studied in cartilage destruction through gene regulation in chondrocytes and therefore were identified as targets for osteoarthritis, a degenerative arthritis which affects joint tissues [145].

The factor *TCF4* present in the network of N’Dama, belongs to a basic helix-loop-helix family which plays an integral role in Wnt signalling and neuronal differentiation especially in the brain development [146,147,148,149,150]. Furthermore, *TCF4* is also involved in the immune responses through the production of plasmacytoid Dendritic Cells (pDCs), which respond to viral nucleic acids and autoimmune diseases, by the secretion of cytokines such as type I interferons [151,152,153,154]. Genetic alterations in *TCF4* are easy targets and therefore mutations in *TCF4* have been reported in the most common form of lymphoma which is the diffuse large B-cell lymphoma and Angelman syndrome [155,156]. The factor *TCF4* has also been identified as the master regulator of schizophrenia, a severe complicated mental disorder [157]. It is reported that disruption in *TCF4* regulatory networks is associated with neuropsychiatric diseases namely schizoprenia, autism, the Pitt-Hopkins syndrome, and depression [158,159]. In connection with AAT, the cattle suffers from fever, listlessness, oedema, depression, and paralysis during the progressive stages of the disease [3,160].

Similar to the networks of liver and spleen tissues, the cooperation network of lymph node tissue reveals the significance of the preferential partner choice of the factor *DBP* and, additionally, it provides a hint that the circadian rhythm in lymph node tissue could be associated with the generation of immune responses, which also includes inflammatory cytokines and the regulation of lipid droplets during the AAT disease.

## 4. Conclusions

Knowledge about TFs and their complex interplay is pivotal to understand the regulation of genetic programs which maintain adaptation of the animal to different pathophysiological stresses like parasitic infections. Our findings indicate that given the AAT disease progression, the preferential partner choice of TFs is strongly related to the tissue type and the susceptibility/resistance of the cattle breeds. Especially the results emphasized the higher relevance of the factor *DBP* along with its partners in circadian rhythm and lipid metabolism, which could be associated with the pathogenesis of AAT in trypanotolerant N’Dama and trypanosusceptible Boran. Importantly, the recent study of Solis et al. [28] on the crucial role of circadian regulation for the coordination of the immune functions lend support to our findings that the circadian control of the immune system influenced by host-pathogen interaction might have resulted in the transcriptional reprogramming of regulation determining the level of AAT-tolerance of the cattle. To the best of our knowledge, this is the first study in this field which mainly focuses on the importance of TFs and their cooperation to reveal the genetic programs underlying the AAT disease. Our results could be used in future works for deciphering the master regulators which could support experimental studies in generating novel hypotheses for potential drug targets.

## Figures and Tables

**Table 1:** Numbers of statistically significant Monotonically Expressed Genes in ascending and descending order for liver-, spleen- and lymph node-tissues for the cattle breeds Boran and N’Dama.

	Boran		N’Dama	
	Ascending	Descending	Ascending	Descending
Liver	741	308	757	124
Spleen	669	126	13	139
Lymph node	87	5	119	114

**Table 2:** Cooperative TF pairs specific for liver-, spleen-, and lymph node tissues of Boran and N’Dama obtained from PC-TraFF approach.

	Cooperative Transcription Factor Pairs		
Breed	Liver	Spleen	Lymph Node
**Boran**	*PPARG–RFX5* *HAND1E47–THAP1* *HAND1E47–TFAP2A* *THAP1–E2F1* *HOXA6–BATF* *TTF1–RFX5* *DBP–PAX8* *E2F1–PPARA* *E2F1–E2F1*	*EMX2–BATF* *FOXM1–JUND* *HOXA4–HOXB7* *PPARA–E2F1* *E2F1–E2F1* *E2F1–TFAP2A*	*JUND–BATF* *HOXA6–MAFF* *SIX3–MAFF* *HAND1E47–E2F1* *HOXA4–BATF* *FOXM1–HNF4G* *DBP–FOXM1* *SIX3–RFX5* *HAND1E47–FOSL1* *FOXA1–MAFF* *HOXA4–MAFF* *SIX3–BATF* *SIX3–HNF4G* *HOXA4–SIX3* *THAP1–PPARA* *E2F1–PPARA* *EMX2–BATF* *THAP1–THAP1*
**N’Dama**	*PPARG–SIX5* *FOXM1–DLX3* *HAND1E47–E2F1* *HAND1E47–USF2* *SIX5–PPARA* *SIX3–THAP1* *THAP1–THAP1* *PPARA–DBP* *PPARA–TFAP2A* *E2F1–E2F1*	*HMBOX–BATF* *HOXB7–BATF* *SIX5–THAP1* *HOXA4–HMBOX1* *HAND1E47–DBP* *HOXA6–BATF* *SIX5–E2F1* *HMBOX1–RFX5* *E2F1–TFAP2A*	*SMAD4–E2F1* *HOXA6–BATF* *HOXA4–HOXB7* *TCF4–HNF4G* *E2F1–TFAP2A* *THAP1–HNF4G* *DBP–TFAP2A* *HAND1E47–RFX5* *THAP1–PPARA* *E2F1–PPARA* *EMX2–BATF* *HOXA4–SIX3* *THAP1–THAP1*

## Supplementary Materials

The following are available online at https://www.mdpi.com/2076-393X/8/2/246/s1, Table S1: The library of non-redundant position weight matrices (PWMs) used in our study, Table S2: Lists of Monotonically Expressed Genes obtained from the MFSelector approach.
